# Pilot genome-wide study of tandem 3′ UTRs in esophageal cancer using high-throughput sequencing

**DOI:** 10.3892/mmr.2014.2003

**Published:** 2014-03-04

**Authors:** MINGZHONG SUN, HUIXIANG JU, ZHONGWEI ZHOU, RONG ZHU

**Affiliations:** Department of Clinical Laboratory, Affiliated Yancheng Hospital, School of Medicine, Southeast University, Yancheng, Jiangsu 224001, P.R. China

**Keywords:** tandem 3′ UTR, esophageal carcinoma, polyadenylation profile, sequencing alternative polyA sites

## Abstract

Regulatory regions within the 3′ untranslated region (UTR) influence polyadenylation (polyA), translation efficiency, localization and stability of mRNA. Alternative polyA (APA) has been considered to have a key role in gene regulation since 2008. Esophageal carcinoma is the eighth most common type of cancer worldwide. The association between polyA and disease highlights the requirement for comprehensive characterization of genome-wide polyA profiles. In the present study, global polyA profiles were established using the sequencing APA sites (SAPAS) method in order to elucidate the interrelation between 3′ UTR length and the development of esophageal cancer. PolyA profiles were analyzed in squamous cell carcinoma, with ~903 genes identified to have shortened 3′ UTRs and 917 genes identified to use distal polyA sites. The genes with shortened 3′ UTRs were primarily associated with adherens junctions and the cell cycle. Four differentially expressed genes were also found, among which three genes were observed to be upregulated in cancerous tissue and involved in the positive regulation of cell motion, migration and locomotion. One gene was found to be downregulated in cancerous tissue, and associated with oxidative phosphorylation. These findings suggest that esophagitis may have a key role in the development of esophageal carcinoma. Furthermore, the genes with tandem 3′ UTRs and differential expression identified in the present study may have the potential to be used as biomarkers for the diagnosis and prognosis of esophageal cancer.

## Introduction

Esophageal carcinoma is a leading cause of cancer-associated mortality. Esophageal carcinoma affects >450,000 individuals worldwide and the incidence of the disease is increasing rapidly. Each year >300,000 new cases of esophageal cancer are reported and various studies have determined the five-year survival rate to be 5–25% (1–8). Esophageal carcinoma is characterized by rapid development and fatal prognosis and is diagnosed at a ratio of ~3–5:1 in males and females, respectively, and has the highest incidence in individuals between 50 and 70 years of age (5,9). There are two main types of esophageal carcinoma: Squamous cell carcinoma (SCC) and adenocarcinoma (ADC). Although SCC and ADC develop from distinct epithelial subtypes, they are associated with chronic inflammation with different aetiologies. Inflammation causes somatically inherited genetic mutations which alter cell cycle control, DNA replication and apoptosis, which result in autonomous and uncontrolled cell proliferation (10). SCC is the predominant form of esophageal carcinoma worldwide. An understanding of the pathogenesis of SCC may give information enhancing early diagnostic and therapeutic strategies.

Whole genome DNA microarrays have been used to identify gene expression profiles and differential gene expression (11–13). Esophageal carcinogenesis is associated with numerous molecular pathways, including those involved in G_1_-phase cell cycle control, cytogenetics, immune evasion and microsatellite instability. Cell cycle regulators, growth factor receptors, adhesion molecules, apoptotic regulators and oncogenes have also been associated with esophageal carcinogenesis (14).

Regulatory regions within the 3′ untranslated region (UTR) influence polyadenylation (polyA), translation efficiency, localization and stability of mRNA. Alternative polyA (APA) has been considered to have a key role in gene regulation since 2008 (15). The differential usage of tandem 3′ UTRs has an important role in gene expression through influencing mRNA stability, transport and translation, generally through the loss and gain of regulatory motifs, particularly microRNA (miRNA)-binding sites (16,17). AU-rich (18) and GU-rich (19) elements are additional cis-elements located at the 3′ UTR of mRNA. PolyA-Seq revealed that ~70% of human genes undergo APA (20). In cancer, a greater number of genes use the proximal polyA site, which leads to high expression of isoforms with shortened 3′ UTRs (21) In addition, it has recently been identified that the shortening of 3′ UTRs has a greater association with proliferation than with transformation induced by oncogene activation (22). In a recent study, a sequencing APA sites (SAPAS) method was used to identify tandem APA site profiles in nasal polyp tissue and paired mucosa of the uncinate process in patients with eosinophilic chronic rhinosinusitis with nasal polyps. In the study, the longer and shorter isoforms were equally represented, implying that 3′ UTR switching in the nasal polyp tissue differs from that in transformed or highly proliferative cells (23).

Few studies have profiled the APA sites in esophageal carcinoma tissue, and the role of polyA in the pathology of esophageal carcinoma is yet to be elucidated. In the present study, tandem APA sites were profiled in a genome-wide fashion using an SAPAS method based on second-generation sequencing technology. A large quantity of tandem APA sites, including genes with significantly differential 3′ UTR length and expression, were identified in esophageal carcinoma. Furthermore, the results were validated using quantitative polymerase chain reaction (qPCR) in an additional eight patients.

## Materials and methods

### Sample preparation and SAPAS sequencing

Normal and cancerous esophageal tissue was obtained from one patient with stage II SCC through esophageal cancer radical surgery at the Third People’s Hospital, (Yancheng, China). Normal tissue adjacent to the esophageal cancer was also obtained, and following pathological examination, was identified to exhibit no cancer lesions. Informed written consent was obtained. Total RNA was extracted from the normal and cancerous esophageal tissue using a TRIzol^®^ kit (no. 15596–026; Invitrogen Life Technologies, Carlsbad, CA, USA). The quantity and quality of the total RNA was assessed using a NanoDrop^®^ ND-1000 spectrophotometer and an RNA Nano 6000 chip on an Agilent 2100 Bioanalyzer (Agilent Technologies, Santa Clara, CA, USA). Two libraries were constructed according to Fu *et al* (24) and sequenced using an Illumina^®^ Genome Analyzer IIx system (Illumina, Inc., San Diego, CA, USA). Normal and cancerous tissue was obtained from a further eight patients and was prepared for qPCR analysis. The immunohistochemical and clinical data of the patients are shown in Table I.

Off-Line Base Caller v1.8 software (Illumina, Inc.) was used to perform the primary analysis of the image generated using the sequencing procedure. FastQC v0.10.1 software was used to estimate the base quality of each lane. Reads with low quality and polyNT (the fragments with a series of single bases, particularly T bases) were filtered. Long reads with poly-T were obtained. Bowtie-indexes were constructed prior to mapping the reads to the human genome hg19 (25) using Bowtie v0.12.7 (26). Unique mappable reads were selected and internal priming reads were filtered. Internal priming reads were reads which mapped to the region within 20 nucleotides (nt) downstream of genomic sequences containing 12 ‘A’ or each of 5′-AAAAAAAA-3′ or 5′-GAAAA+GAAA+G-3′, which were regarded as disruption sequences due to their ability to bind to primers with their A-rich genomic regions, but not with the polyA tail. Statistical analyses were performed using the number prior to and following filtering and mapping.

### APA annotation and statistical analysis of tandem 3′ UTRs

As described by Tian *et al* (27), the sequences were clustered according to their position on the 3′ UTR. For example, if two reads map to the same strain of the same chromosome, and their positions on the 3′ UTR are within 24 nt, various 3′ end cleavage clusters may be formed. Each cleavage cluster with more than one read was defined as a polyA site. The polyA sites were annotated as University of California, Santa Cruz (UCSC), Tian polyA_DB and novel sites using gene annotation information, including Known Genes (25) and polyA_DB2 (28), from the UCSC Genome browser. The annotation procedure was as follows: (i) PolyA sites located within 24 nt of the 3′ UTR of the UCSC transcripts were annotated as UCSC; (ii) PolyA sites located within 24 nt of the 3′ UTR of polyA_DB2 were annotated as Tian polyA_DB and (iii) The remaining polyA sites were annotated as novel sites. According to their different locations across the genome, all these sites were separated and classified as ≤1 k nt downstream and classified as 3′ UTRs, coding DNA sequences, intergenic sequences, introns and noncoding genes. The number of polyA sites was calculated using the number of reads and genes.

Genes containing two or more tandem polyA sites were defined as tandem 3′ UTR genes. The annotated polyA sites in the two samples were compared for further analysis. Linear trend tests were performed for tandem APA switch testing of genes containing two or more tandem polyA sites which were expressed in the two samples. In brief, 3′ UTR length was calculated for each tandem polyA site. A column chain table was then generated and the number of reads in each tandem polyA site was recorded for the two samples, which was regarded as the expression level. The tandem polyA sites were considered as a column, from the polyA site with the shortest 3′ UTR to that with the longest 3′ UTR. The samples were regarded as a row, with sample one in row one and sample two in row two. If the total number of reads in the table was <30, then the gene was not included. The length of the 3′ UTR was defined as the height of the column, with the width of the row for sample one recorded as one and that for sample two recorded as two. The

$$\text{r}=\frac{\text{cov}(\text{X},\text{Y})}{\sigma_{\text{X}}\sigma_{\text{Y}}}=\frac{\text{E}((\text{X}-\mu_{\text{X}})(\text{Y}-\mu_{\text{Y}}))}{\sigma_{\text{X}}\sigma_{\text{Y}}}=\frac{\text{E}(\text{XY})-\text{E}(\text{X})\text{E}(\text{Y})}{\sqrt{\text{E}({\text{X}}^{2})-{\text{E}}^{2}(\text{X})}\sqrt{\text{E}({\text{Y}}^{2})-{\text{E}}^{2}(\text{Y})}}$$

number of reads in the table was considered as a set value, and the width of the row or column was considered to be a coordinate. The Pearson correlation coefficient r was then calculated (in short for tandem APA sites switch index, TSI). The formula for the Pearson correlation coefficient r is as follows:

The χ^2^ distribution with one degree of freedom was calculated for samples >30 using the following formula: M^2^=(n-1)r^2^. P-values were then calculated, as were false discovery rates (FDRs) using the Benjamini-Hochberg method.

For genes with a low FDR, a TSI>0 indicates that the 3′ UTR of the gene in sample two was lengthened. However, a TSI<0, suggests that the 3′ UTR of the gene in sample two was shortened. Genes with r<−0.1 and FDR<0.01 were considered to be shortened 3′ UTR genes, while genes with r>0.1 and FDR<0.01 were considered to be lengthened 3′ UTR genes. R software was used to generate a scatter plot illustrating the correlation between lengthened 3′ UTR genes and shortened 3′ UTR genes, and the relevant coefficient was calculated. The number of lengthened 3′ UTR genes and shortened 3′ UTR genes were calculated separately and the χ^2^ test was performed.

### Differentially expressed genes (DEGs) and functional enrichment

SAPAS solely sequenced the 3′ UTRs; therefore, the length of each gene is able to be disregarded when estimating the gene expression level. Thus, the read number mapped to a gene represents the expression level of that gene. The expression of each gene was assessed as described previously by Audic and Claverie (29). Using the Benjamini-Hochberg method, the P-value was then adjusted. Genes with FDR<0.01 were considered to be DEGs. DAVID Bioinformatics Resources 6.7 (30) was used to perform functional annotation and enrichment of the tandem 3′ UTR genes and the DEGs separately.

### qPCR analysis

Total RNA was extracted using TRIzol^®^ (Invitrogen Life Technologies). For each sample, 100 ng total RNA was used in reverse transcription reactions using oligo-dT primers and the SuperScript^®^ III Reverse Transcriptase (Invitrogen Life Technologies). For each gene, two gene-specific primer sets were designed according to Tian *et al* (23). qPCR analysis was performed using the Light Cycler 480 instrument (Roche Biochemicals, Indianapolis, IN, USA). The expression ratio of the shortened region to the lengthened region was represented by the ratio of the constitutive UTR (cUTR) to the extended UTR (eUTR), and was calculated using the 2^−ΔΔCt^ value for each gene by normalizing the eUTRs to the cUTRs. For the DEGs, relative gene expression was quantified in the cancerous tissue using GAPDH as an endogenous control.

## Results

### Sample info and sequencing procedure

In the normal and cancerous tissue, 54.3 and 39.7 μg total RNA was extracted, respectively. Furthermore, NanoDrop analysis revealed that the the optical density (OD_260/280_) values of the normal and cancerous tissues were 1.92 and 1.89, respectively. The RNA integrity numbers assessed using the Agilent Bioanalyzer 2100 instrument were 7.2 and 7.7 in the normal and cancerous tissue, respectively, suggesting that the total RNA in the two samples was suitable for the subsequent experiments. Following library construction, analysis using the Agilent Bioanalyzer 2100 instrument revealed that the length of the two libraries was ~350 bp (range, 250–500 bp).

### Primary analysis of the sequencing data

APA sites were analyzed in one patient with esophageal carcinoma using the SAPAS method (24). Normal and cancerous tissue samples were sequenced using the Illumina Genome Analyzer IIx platform. The sequencing data were uploaded to the Sequence Read Archive database at the National Center for Biotechnology Information and are accessible using the accession number SRA100663. The raw reads in the two samples were 23.1 and 16.9 M. Following filtering, the reads were ~21.8 and 15.6 M, which was >92% of the total reads sequenced. Subsequently, 16.2 and 13.0 M reads were mapped to the hg19 human nuclear genome, which were >70% of the total reads. Among these reads, >56% were uniquely mapped to the human nuclear genome. Internal priming reads were then filtered and 8.5 and 7.1 M reads were retained for APA site annotation and tandem APA switching tests.

### APA site annotation

**Figure 1:**
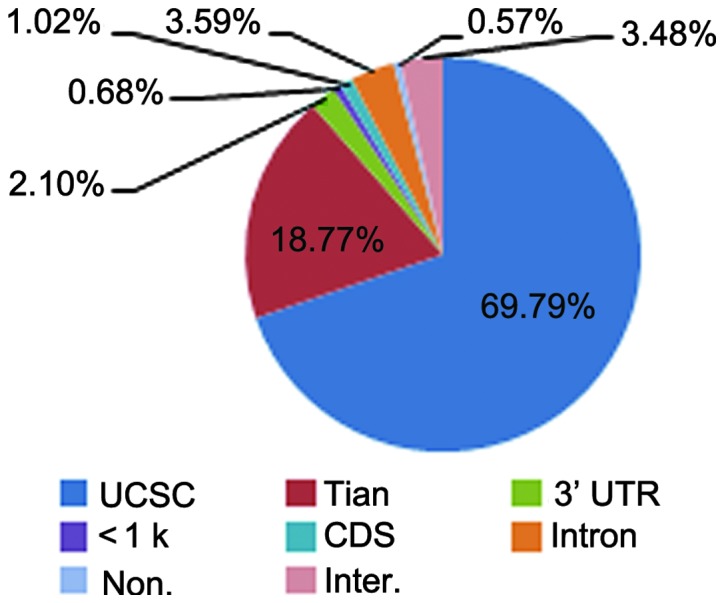
Genomic location of reads which were uniquely mapped to the nuclear genome subsequent to internal priming filtering. UTR, untranslated region; Inter, intergenic; Non, noncoding gene; <1k, <1 knt downstream; Tian: Tian polyA_DB; TES, polyA sites that are located within 24 nt downstream of 3′ transcript end of UCSC canonical genes; CDS, coding DNA sequence.

**Figure 2:**
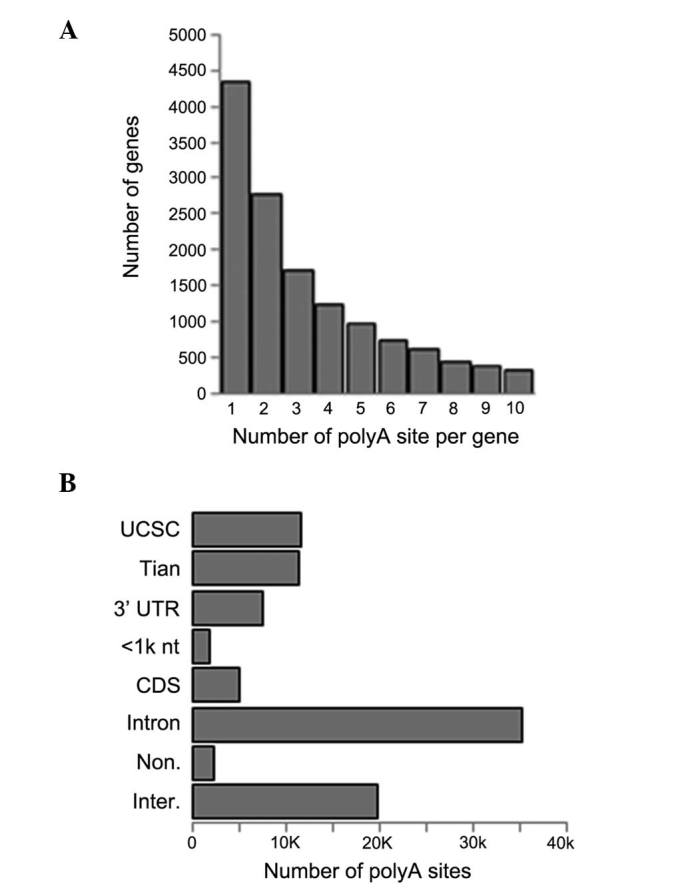
Characteristics of the poly(A) sites of one patient with squamous cell carcinoma. (A) Distribution of the number of poly(A) sites per gene. (B) Genomic location of the poly(A) sites in all genes. UTR, untranslated region; Inter, intergenic; Non, noncoding gene; <1 knt, <1 knt downstream; Tian, Tian polyA_DB; TES, polyA sites that are located within 24 nt downstream of 3′ transcript end of UCSC canonical genes; CDS, conding DNA sequence.

The APA sites of 15,186 and 14,486 genes (Table II) were annotated in the normal and cancerous tissue samples, respectively. Subsequent to filtering the polyA sites supported by solely one read, 14,597 and 13,658 genes with polyA sites were obtained for the normal and cancerous tissue, respectively. ~88.6% of the reads were mapped to the region within 24 nt of the known polyA sites. Furthermore, 2.1 and 0.7% of the reads mapped to the 3′ UTR region of UCSC (http://genome.ucsc.edu/) canonical gene and 1 kb downstream of the end of the genes, respectively (Fig. 1). In the normal tissue, 10,850 genes (71.4%) were annotated with more than one polyA site and 8,072 genes (54.2%) were annotated with more than two polyA sites (Fig. 2A). The reads distribution for the number of polyA sites within 10 is shown in Fig. 1. Among the 75,257 annotated polyA sites in the normal tissue, 24.3% of the sites were recorded in the polyA_DB2 database (28). This suggests that a greater number of novel polyA sites are detected in the genome using the SAPAS method, particularly those in lowly expressed mRNA (Fig. 2B).

### Tandem polyA site analysis

**Figure 3:**
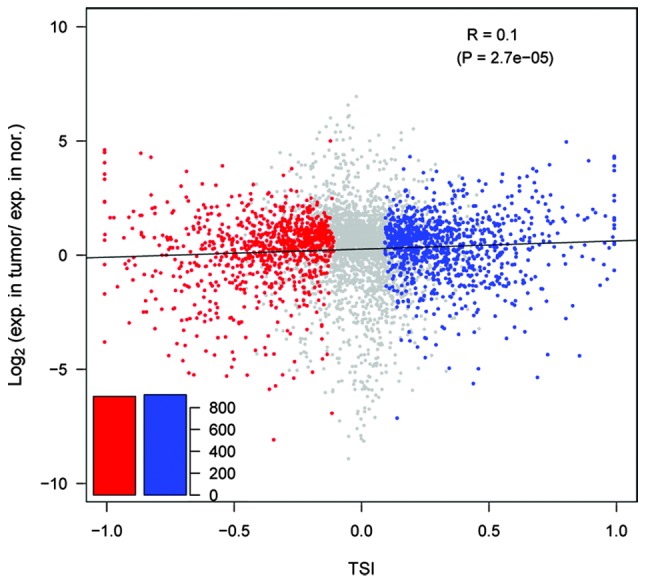
APA site switching and gene expression in normal and cancerous tissue. TSI values are plotted against the Log_2_ of the expression level of genes from the cancerous tissues relative to normal tissues. Larger TSI values indicate that longer tandem UTRs are present in carcinoma tissues. Genes with significant switching to longer (blue) or shorter (red) tandem UTRs in cancerous tissues are colored (false discovery rate<0.01). Nor, normal tissue; Exp, expression; TSI, tandem APA site switch index; UTR, untranslated region. APA, alternative polyadenylation.

A total of 1,820 genes was identified to show a significant difference in tandem 3′ UTR length (FDR<0.01). Among these genes, 50.4% (903) had a negative TSI, suggesting that the number of genes with shortened 3′ UTRs in the cancerous tissue was approximately equal to the number of those with lengthened 3′ UTRs (Fig. 3). The shortened genes in the cancerous tissue were enriched in 119 gene ontology (GO) terms primarily associated with protein transport and the establishment of protein localization (FDR<0.01; data not shown). The cell components encoded by these GO term-enriched genes were associated with membrane-enclosed lumens and the internal side of the plasma membrane (FDR<0.01). All genes were enriched in the following 11 pathways: Chronic myeloid leukemia, adherens junction, SNARE interactions in vesicular transport, cyanoamino acid metabolism, notch signaling, T-cell receptor signaling, cell cycle, spliceosome, biosynthesis of unsaturated fatty acids, Fcγ receptor-mediated phagocytosis and nitrogen metabolism (Table III). The lengthened genes in cancerous tissues were enriched in 141 GO terms, which were associated with one biological process: Intracellular transport (FDR<0.01; data not shown). All genes were enriched in the following six pathways: Lysosome, nucleotide excision repair, wingless-type MMTV integration site family signaling, ubiquitin mediated proteolysis, porphyrin and chlorophyll metabolism and spliceosomes (Table IV).

**Figure 4:**
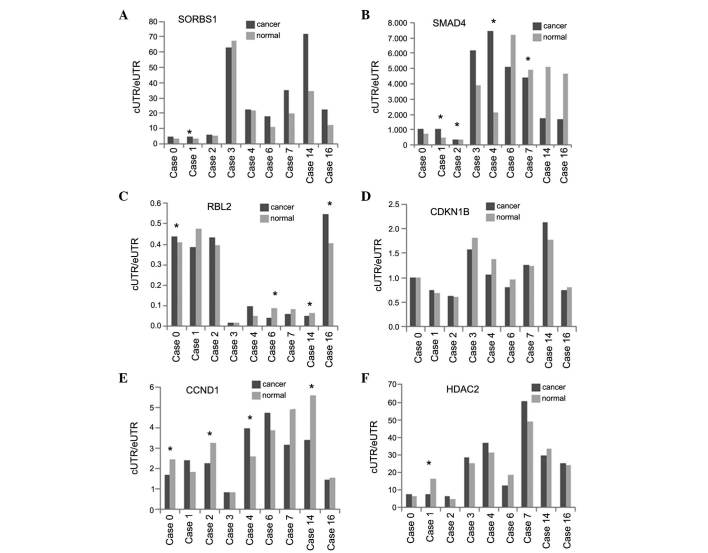
Quantitative polymerase chain reaction analysis of tandem 3′ UTR genes in nine patients with esophageal cancer. (A) SORBS1, (B) SMAD4, (C) RBL2, (D) CDKN1B, (E) CCND1 and (F) HDAC2. cUTR/eUTR represents the expression ratio of of the shortened region to the lengthened region. ^*^P<0.05. UTR, untranslated region; cUTR, constitutive UTR; eUTR, extended UTR; SORBS1, sorbin and SH3 domain containing 1; SMAD4, SMAD family homolog 4; RBL2, retinoblastoma-like 2; CDKN1B; cyclin-dependent kinase inhibitor 1B; CCND1, cyclin D1; HDAC2, histone deacetylase 2.

Seven genes with significantly shortened tandem 3′ UTRs were selected for qPCR analysis. Six of the seven genes were successfully verified in more than four patients, including the patient used for SAPAS sequencing (Fig. 4). The seven genes were as follows: Sorbin and SH3 domain containing 1 (SORBS1), SMAD family member 4 (SMAD4), retinoblastoma-like 2 (RBL2), cyclin-dependent kinase inhibitor 1B (CDKN1B), cyclin D1 (CCND1) and histone deacetylase 2 (HDAC2). SORBS1 was enriched in adherens junctions, SMAD4 was enriched in adherens junctions and the cell cycle, RBL2 and CDKN1B were enriched in the cell cycle and HDAC2 was enriched in the notch signaling pathway and the cell cycle.

### Analysis of DEGs

A total of 5,911 genes was identified to be differentially expressed. Among these genes, 2,168 were upregulated by two-fold in the cancerous tissue compared with those in the normal tissue (FDR<0.01), while 3,743 genes were downregulated. The upregulated genes were enriched in two GO terms, primarily involved in the positive regulation of cell motion and migration (FDR<0.01) (Table V). No Kyoto Encyclopedia of Genes and Genomes (KEGG) pathways were enriched for the upregulated genes. The downregulated genes were enriched in three GO terms associated with translational elongation, translation and mitochondrion organization (FDR<0.01; Table V). These enriched genes encode cytosolic ribosome and mitochondrial cell components, which function in ribosome structure, nucleotide binding, GTPase activator activity and enzyme binding. These genes are primarily enriched on the following five KEGG pathways: Ribosomes, Alzheimer’s disease, oxidative phosphorylation, Parkinson’s disease and Huntington’s disease (Table VI).

**Figure 5:**
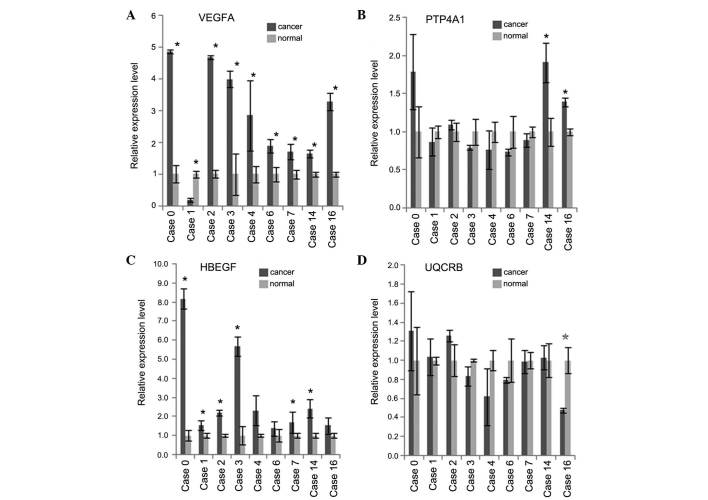
Quantitative polymerase chain reaction analysis of differentially expressed genes in nine patients with esophageal cancer. (A) VEGFA, (B) HBEGF, (C) PTP4A1, (D) UQCRB. ^*^P<0.05. VEGFA, vascular endothelial growth factor A; HBEGF, heparin-binding EGF-like growth factor; PTP4A1, protein tyrosine phosphatase type IVA; UQCRB, ubiquinol-cytochrome c reductase binding.

Three upregulated DEGs, vascular endothelial growth factor (VEGF) A, heparin-binding EGF-like growth factor (HBEGF) and protein tyrosine phosphatase type IVA (PTP4A1), and one downregulated DEG, ubiquinol-cytochrome c reductase binding (UQCRB), were confirmed in more than four patients (Fig. 5). VEGFA, HBEGF and PTP4A1 were identified to be upregulated 2–4-fold in cancerous tissue and enriched in the positive regulation of cell motion, migration and locomotion. UQCRB was found to be downregulated 4-fold in cancerous tissue and to be enriched in the process of oxidative phosphorylation.

## Discussion

APA site switch events have been reported in numerous human diseases, including breast (24), colon (31), lung (31), kidney (21) and liver cancer (21). A previous study suggested the association of shortened 3′ UTRs with gene regulation and cancer development (31). In the present study, APA site profiles were generated in the normal and cancerous tissue of one patient with esophageal cancer using the SAPAS method. ~903 genes were identified to have shortened 3′ UTRs and 917 genes were found to use distal polyA sites. In the present study, the number of lengthened genes was observed to be similar to the number of shortened genes, which is different to the trend found in other tumor tissues. A genome-wide study revealed that 3′ UTR switching was different in nasal polyp tissue to that in transformed or highly proliferative cells. These findings suggest that the fundamental mechanism underlying polyA site switching in the development of esophageal carcinoma may be more similar to the process of inflammation than to that in a tumor. Chronic inflammation is a significant risk factor for the development of cancer (32) and a correlation has been identified between reflux-induced inflammation and esophageal carcinogenesis (33). Moreover, basement membrane changes induced by chronic inflammation are associated with aberrant proliferation in esophageal epithelia (34). Therefore, the development of esophageal carcinoma may be largely based on esophagitis and cancer-associated factors should be elucidated in early esophagitis. More research on esophagitis and esophageal carcinoma is required in order to validate this hypothesis.

In the present study, in order to confirm the SAPAS findings, six shortened tandem 3′ UTR genes, SORBS1, SMAD4, RBL2, CDKN1B, CCND1 and HDAC2, were assessed using qPCR analysis. These genes were enriched in adherens junction, cell cycle and notch signaling pathways, all of which are cancer-associated pathways (10,35,36). SORBS1 has been found to be enriched in adherens junction-associated pathways and is regarded as an obesity gene (37). Reports have shown that obesity and being overweight are risk factors for the development of esophageal cancer (38). SMAD4 is enriched in adherens junction and cell cycle pathways and has been found to have a potential predictive value for esophageal squamous cell carcinoma in patients receiving neoadjuvant chemoradiotherapy (39) RBL2 and CDKN1B are enhanced in cell cycle pathways. A cell cycle-associated oncogene, CCND1, has been reported to contain two miRNA binding sites for miR-15 and -16 (31) HDAC2 in enhanced in notch signaling and cell cycle pathways. Furthermore, among the six genes, SMAD4 and CCND1 have been considered as molecular markers in esophageal squamous cell carcinoma (40). These findings suggest that the shortening of the six candidate genes may be, at least in part, associated with the molecular pathology of esophageal cancer development. However, more research is required to further elucidate the interrelation between these candidate genes and esophageal cancer.

In addition, four DEGs which were associated with the positive regulation of cell motion, migration and locomotion, were selected. VEGFA, HBEGF and PTP4A1 were found to be upregulated, while UQCRB was observed to be downregulated, in the cancerous tissue compared with the normal tissue. qPCR analysis has previously revealed that VEGFA is upregulated in the HN-Eso-1 metastatic esophageal spindle cell carcinoma-derived cell line. VEGFA expression was reported to be involved in the HN-Eso-1 cell autocrine system and to have a protective effect against anti-tumor agents (41). HBEGF is a member of the epidermal growth factor family. The membrane-bound proHB-EGF is a precursor of the soluble form of HB-EGF (sHB-EGF), which promotes cell proliferation and survival (42). The PTP4A1 protein is a cell signaling molecule that has regulatory roles in a variety of cellular processes, including cell proliferation and migration. PTP4A1 may also be involved in cancer development and metastasis (http://www.ncbi.nlm.nih.gov/gene/7803). In the present study, in the nine samples used for qPCR analysis, the regulational role of PTP4A1 was found to be highly associated with p27, suggesting that PTP4A1 upregulation may be associated with the prognosis of esophageal cancer. In the present study, UQCRB was observed to be downregulated four-fold in the cancerous tissue compared with the normal tissue, and enriched in oxidative phosphorylation. This mitochondrial oxidative phosphorylation gene is considered to be a prognostic marker for colorectal cancer (43). The evidence above suggests with further investigation, that the DEGs analyzed in the present study may be used as biomarkers for the diagnosis and prognosis of esophageal cancer.

The present study generated genome-wide polyadenylation profiles using the SAPAS method in order to elucidate the interrelation between 3′ UTR length and esophageal cancer development. Polyadenylation profiles were generated for one patient with SCC and several candidate biomarkers were validated in a further eight patients with esophageal cancer using qPCR analysis. In conclusion, APA site switching may be associated with esophageal cancer development and prognosis. Furthermore, these findings may be applied to other tumor types.

## Figures and Tables

**Table I:** Immunohistochemical and clinical characteristics of nine patients with esophageal carcinoma used for sequencing alternative polyadenylation sites and quantitative polymerase chain reaction analysis.

Case #	Age/Gender	Neu	EGFR	TOPII	P53	Ki67 (%)	PCNA	P21	GST-π	c-myc	P27
0^a^	52/M	−	+	−	−	5	++	+	+	−	++
1^b^	66/M	−	−	−	−	5	+	−	+	−	−
2^b^	66/M	++	+	+	−	20	++	+	++	−	+
3^b^	65/M	++	−	−	++	5	++	−	+	−	−
4^b^	74/F	++	−	−	−	−	++	−	+	−	−
6^b^	59/F	+	+	−	−	5	++	−	+	−	−
7^b^	68/M	+	+	+	−	20	+	−	+	−	−
14^b^	72/F	−	−	−	++	5	++	−	+	−	+
16^b^	56/M	−	−	+	−	30	++	−	+	−	+

a Samples used for sequencing;

b Samples used for qPCR analysis.

M, male; F, female; EGFR, epidermal growth factor receptor; Neu, neuro/glioblastoma derived oncogene homolog; TOPII, DNA topoisomerase II; PCNA, proliferating cell nuclear antigen; GST, glutathione *S*-transferase.

**Table II:** Summary of statistical analyses of sequencing alternative polyadenylation site data obtained from Illumina Genome Analyzer IIx sequencing.

Parameter	Normal tissue	Cancerous tissue
Raw reads	23,059,686	16,903,581
Clean reads	21,807,523 (94.6%)	15,623,947 (92.4%)
Mapped to genome	16,229,331 (70.4%)	12,990,709 (76.9%)
Uniquely mapped to genome	13,064,869 (56.7%)	10,405,340 (61.6%)
Mapped to nuclear genome	12,925,041 (56.1%)	10,188,625 (60.3%)
Passed internal priming filter	8,543,416 (37.1%)	7,056,710 (41.8%)
Genes sampled by reads	15,186	14,486
Poly(A) sites	75,257	72,619
Known poly(A) sites sampled	20,196	18,894
Putative novel poly(A) sites	55,061	53,725
Genes sampled by poly(A) sites	14,597	13,658

Poly(A), polyadenylation.

**Table III:** Enrichment of KEGG pathways in genes with shortened 3′ untranslated regions.

KEGG ID	KEGG name	List hits	List size	Pop hits	Pop size	Enrichment fold	P-value	FDR
hsa05220	Chronic myeloid leukemia	9	248	75	5085	2.46	2.80×10^−2^	2.90×10^1^
hsa04520	Adherens junction	9	248	77	5085	2.40	3.22×10^−2^	3.26×10^1^
hsa04130	SNARE interactions in vesicular transport	6	248	38	5085	3.24	3.52×10^−2^	3.50×10^1^
hsa00460	Cyanoamino acid metabolism	3	248	7	5085	8.79	4.20×10^−2^	4.04×10^1^
hsa04330	Notch signaling pathway	6	248	47	5085	2.62	7.59×10^−2^	6.14×10^1^
hsa04660	T-cell receptor signaling pathway	10	248	108	5085	1.90	7.80×10^−2^	6.24×10^1^
hsa04110	Cell cycle	11	248	125	5085	1.80	8.12×10^−2^	6.39×10^1^
hsa03040	Spliceosome	11	248	126	5085	1.79	8.46×10^−2^	6.55×10^1^
hsa01040	Biosynthesis of unsaturated fatty acids	4	248	22	5085	3.73	8.83×10^−2^	6.72×10^1^
hsa04666	Fcγ receptor-mediated phagocytosis	9	248	95	5085	1.94	8.91×10^−2^	6.75×10^1^
hsa00910	Nitrogen metabolism	4	248	23	5085	3.57	9.81×10^−2^	7.12×10^1^

KEGG, kyoto encyclopedia of genes and genomes; hsa, *Homo sapiens*; SNARE, SNAP receptor; SNAP, Soluble *N*-ethylmaleimide-sensitive fusion protein attachment protein; FDR, false discovery rate; Pop, population.

**Table IV:** Enrichment of KEGG pathways in the genes with lengthened 3′ untranslated regions.

KEGG ID	KEGG name	List hits	List size	Pop hits	Pop size	Enrichment fold	P-value	FDR
hsa04142	Lysosome	15	240	117	5085	2.72	1.06×10^−3^	1.27
hsa03420	Nucleotide excision repair	7	240	44	5085	3.37	1.60×10^−2^	1.77×10^1^
hsa04310	Wnt signaling pathway	13	240	151	5085	1.82	5.07×10^−2^	4.68×10^1^
hsa04120	Ubiquitin mediated proteolysis	12	240	137	5085	1.86	5.64×10^−2^	5.05×10^1^
hsa00860	Porphyrin and chlorophyll metabolism	5	240	33	5085	3.21	6.69×10^−2^	5.68×10^1^
hsa03040	Spliceosome	11	240	126	5085	1.85	7.13×10^−2^	5.92×10^1^

KEGG, kyoto encyclopedia of genes and genomes; hsa, *Homo sapiens;* FDR, false discovery rate; Wnt, wingless-type MMTV integration site family; Pop, population.

**Table V:** Enrichment of gene ontology biological processes in differentially expressed genes.

GO category	Genes upregulated in cancerous tissue (2168)	Genes downregulated in cancerous tissue (3743)
FDR<0.01	Positive regulation of cell motion (28) Positive regulation of cell migration (26)	Translational elongation (56) Translation (107) Mitochondrion organization (51)
FDR<0.05	Positive regulation of locomotion (27) Regulation of cell motion (41)	RNA processing (146) Oxidative phosphorylation (38) Vesicle-mediated transport (151)
FDR<0.1	Wound healing (40)	

Between normal and cancerous tissues, FDR=0.01 and a greater than two-fold difference exists. Numbers in the brackets indicate the number of genes for the particular category. FDR, false discovery rate.

**Table VI:** Enrichment of KEGG pathways in downregulated differentially expressed genes between cancerous and normal tissues.

KEGG ID	KEGG name	List hits	List size	Pop hits	Pop size	Enrichment fold	P-value	FDR
hsa03010	Ribosome	46	1061	87	5085	2.53	1.32×10^−10^	1.6510^−7^
hsa05010	Alzheimer’s disease	58	1061	163	5085	1.71	1.55×10^−5^	1.94×10^−2^
hsa00190	Oxidative phosphorylation	48	1061	130	5085	1.77	3.30×10^−5^	4.12×10^−2^
hsa05012	Parkinson’s disease	47	1061	128	5085	1.76	4.75×10^−5^	5.93×10^−2^
hsa05016	Huntington’s disease	61	1061	180	5085	1.62	4.84×10^−5^	6.04×10^−2^

KEGG, kyoto encyclopedia of genes and genomes; FDR, false discovery rate; hsa, *Homo sapiens*; Pop, population.
